# On Patient Quality of Life: Impacts of Knee Osteoarthritis on Pain, Anxiety, Depression, Fatigue and Sleep Disorders

**DOI:** 10.1002/nop2.70264

**Published:** 2025-07-03

**Authors:** Guang‐Zhao Li, Rong‐Jian Ji, Cui Ping Xu, Li‐Juan Yang, Paulo Moreira

**Affiliations:** ^1^ Department of Nursing Shandong Provincial Hospital Affiliated to Shandong First Medical University Jinan Shandong China; ^2^ Department of Nursing The First Affiliated Hospital of Shandong First Medical University & Shandong Provincial Qianfoshan Hospital Jinan Shandong China; ^3^ International Healthcare Management Research & Development Center The First Affiliated Hospital of Shandong First Medical University & Shandong Provincial Qianfoshan Hospital Jinan Shandong China; ^4^ Atlantica Instituto Universitario Gestao Em Saude Oeiras Portugal; ^5^ School of Social Affairs, Henan Normal University Xinxiang China

**Keywords:** anxiety, depression, knee osteoarthritis, pain, quality of life, sleep disorders

## Abstract

**Aim:**

Patients repeatedly experiencing a sustained disease course, recurrent chronic pain, and serious sleep disorder will weaken their physical resistance and self‐care ability. Further evidence on patients with knee osteoarthritis (KOA) and related latent classes (LCs) of symptom clusters is needed in order to clarify its significance for symptom management and quality of life (QoL) enhancement.

**Design:**

This study aimed to generate evidence on differences in symptoms experienced by patients with knee osteoarthritis, identify related LCs, and explore the differences of each LC by demographic, disease, individual characteristics, and QoL.

**Methods:**

A convenience sample of 230 participants with knee osteoarthritis was surveyed. Sociodemographic and symptom questionnaires were collected. The analysis focused on the influencing factors that are associated with LC, intergroup differences, and associated QoL impacts.

**Results:**

A total of 230 participants with knee osteoarthritis completed the survey, average age was 64.54 ± 7.59. Three LCs were associated with knee osteoarthritis: LC1 (low symptom class), LC2 (high psychological disorder class) and LC3 (high fatigue with sleep disorder class), accounting for 56%, 24% and 20% respectively. Compared to LC1, LC2 was related to poor economic status, higher imaging grade and lower self‐efficacy. Notably, being older, being less educated, higher occurrence of chronic diseases, experiencing negative life events and having lower self‐efficacy rose in LC3.

**Patient or Public Contribution:**

The study identified LCs according to pain, anxiety, depression, fatigue and sleep disorder symptoms among patients with KOA, providing a foundation for further determining whether the clusters vary over time or along disease and treatment trajectory and what their possible synergistic effects are on KOA and associated QoL.

## 1 Introduction

Knee osteoarthritis (KOA) refers to a chronic degenerative joint disease, which mainly affects articular cartilage, synovium and other surrounding tissue structures, and results in swelling and pain, movement disorders and deformities in the knee joint (Leung 2018). Further, the incidence of KOA is increased in middle‐aged and older adults, and epidemiological surveys have revealed that the prevalence has increased to 8.1% in China (Tang et al. 2016). Remarkably, it is estimated to be the fourth most common debilitating disease until 2020, indicating that this is a growing concern for public health (Liu et al. 2015). Alarmingly, 80% of individuals with KOA experienced physiological role limitations. Moreover, an emerging study showed that 47% of 231 patients with knee or hip arthritis had severe fatigue, which directly affects their enthusiasm to participate in social activities and negatively impacts their quality of life (QoL) (Broeder 2011). Apart from physiological inabilities, KOA may also contribute to a variety of psychological symptoms, such as depression, anxiety and sleep disorders. Regarding depression, Wu Hai revealed that 47.4% of patients with KOA experienced anxiety or depression (Wu et al. 2018). Concerning sleep disorders, a study conducted with 2682 cases of KOA showed that 71% had sleep disorders, triggering pessimistic results on sleep quality (Allen et al. 2008). Sariyildiz et al. found that negative emotions such as anxiety and depression, as well as sleep disorders in patients with knee osteoarthritis, can lead to central sensitisation, which is a phenomenon involving the central nervous system's abnormal sensitivity to pain signals. This enhanced sensitivity can lead to an amplification of pain perception (Sariyildiz et al. 2023). Meanwhile, a lack of sleep makes individuals vulnerable to fatigue, and this aggravates physical and mental symptoms, and eventually impinges on their QoL (Petrov et al. 2015). In summary, patients with knee osteoarthritis have multiple symptoms that exist and affect each other, significantly reducing their QoL.

While some KOA patients will experience one or a few symptoms unaccompanied, the majority will experience multiple symptoms simultaneously. As is well known, earlier studies have mainly focused on exploring isolated symptoms and its relationship with influencing factors. Although such methods can accurately concentrate on matching symptoms, this approach ignores the interaction between multiple symptoms. In addition, a former study suggested that intervention strategies considering multiple concurrent symptoms are superior to those focusing on a single symptom (Lenz et al. 1997); thus, focusing on the symptom clusters of KOA patients is necessary.

Kim explained the symptom clusters as two or more interrelated symptoms that form a stable group and that are independent from other symptoms (Kim et al. 2005). Because of symptom clusters, emerging research transitioned from studying diseases as ‘single symptoms’ to the ‘symptom group’. In addition, current investigations have explored the number of symptoms experienced in chronic diseases equal to that of cancer research, and symptom severity is also worth considering (Bekelman et al. 2009). To our knowledge, current research concerning symptom clusters of chronic disease mainly focuses on chronic obstructive pulmonary disease (Lim et al. 2017), heart failure (Wang 2018), haemodialysis (Zhou 2013), permanent colostomy (Peng 2019) and others, but is limited for KOA patients. Therefore, further research conforms to the trend of chronic disease symptom management but also enriches and deepens the content of this research.

The latent class model (LCM), also called latent class analysis (LCA), is a statistical method that uses intermittent latent variables to explain the correlation between explicit indicators so that the correlation between explicit indicators can be explained by latent variables to maintain local independence (Wang and Bi 2018). The principle of latent profile analysis (LPA) is completely consistent with LCM, except that LPA uses continuous variables, and LCA uses counting observation variables. Therefore, based on the basic principle of LPA, this study divided the best latent class (LC) by pain, anxiety, depression, sleep disorders and fatigue among KOA patients; this was done to provide initial research for the future implementation of small sample homogeneous group interventions and to maximise the intervention effect.

The symptom experience of KOA patients was explored for a variety of reasons (Jenkins and McCoy 2015). However, most studies assume that symptoms remain homogeneous and unified, which goes against the development of personalised intervention programmes for KOA patients. Thus far, only one study has been conducted in a group of 75 KOA patients, divided into two subgroups, ‘low pain‐high depression group’ and ‘symptom balance group’ using cluster analysis (Jenkins and McCoy 2015). Given the cultural and regional differences locally and internationally, the results were not necessarily applicable to China, the sample size was small, and the conclusions obtained by cluster analysis were subjective. Therefore, our study uses the QoL of patients with KOA as an index to measure their state of life. Previous studies (Ji 2017; Mou 2019) have shown that symptom groups can significantly associate with the QoL of patients, and whether this finding applies to KOA patients in different LCs remains unclear. Considering these limitations, our study investigated the individual differences in symptoms experienced among patients with knee osteoarthritis, analysed and identified the LC of symptom clusters and explored the differences of each LC of symptom clusters regarding demographics, diseases, individual characteristics and QoL. To develop this study, we have also considered key trends and recommendations in nursing and healthcare management research which helped focussing this study (Hai et al. 2024; Kehinde et al. 2023; Loureiro Pais Batista et al. 2023).

## 2 Methods

### 2.1 Participants and Design

This cross‐sectional study was carried out in bone and joint wards of two tertiary hospitals in Jinan, Shandong province, China, from March 2019 to June 2019. We identified potential participants by medical record, flyer, and clinician visit list. The selected participants were required to have a certain capacity for communication and understanding, follow the informed consent process, join voluntarily and meet the 2018 guidelines for the diagnosis and treatment of osteoarthritis issued by the Orthopaedic Branch of the Chinese Medical Association KOA diagnostic criteria (Joint Surgery Group, Chinese Society of Osteology, Chinese Medical Association 2018): (a) Experiencing knee joint pain for the majority of the previous month; (b) X‐ray showed osteophytes at the edge of the joint; (c) X‐ray followed the joint fluid examination of osteoarthritis; (d) ≥ 40 years old; (e) Morning stiffness of ≤ 30 min, collected via medical record; (f) Joints had bone friction sounds during activity. The X‐rays were performed for inclusion/exclusion purposes. The KOA diagnostic criteria are satisfied if one of the following combinations is met: a + b, a + c + e + f or a + d + e + f. In our study, the diagnosis of knee osteoarthritis was completed by a professional orthopaedist through X‐ray and symptom presentation. We excluded cases with rheumatic or rheumatoid arthritis, undergoing knee arthroplasty and with a history of fatigue syndrome, as well as severe mental illnesses such as anxiety or depression. In our study, a convenience sampling was used to select patients. According to Kendall's calculation principle of sample size for cross‐sectional investigation studies, the sample size should be 5–10 times the number of independent variables (Hu et al. 2008), and 20 independent variables are included in our study. Considering a 15% questionnaire loss rate and unqualified rate in the process of data collection, the sample size should be expanded to 115–230 cases. This study strictly followed the maximum sample size estimation standard, and the final sample size was 230 cases. The number of patients approached was 254, eligible was 242 and 242 patients agreed to participate. In total, 242 questionnaires were collected, including 12 questionnaires with more than 10% missing data and 230 valid questionnaires. This study has been approved by the Ethics Committee of REDACTED.

### 2.2 Measurements

#### 2.2.1 General Description of Sample

Sociodemographic included age, gender, residence, education, marital status and economic and living style. We collected clinical information from the hospital information system (HIS), which comprised body mass index, position of the knee, disease course, chronic complications and imaging grading. Self‐efficacy and negative life events were obtained through self‐reported questionnaires.

#### 2.2.2 Numerical Rating Scale

The numerical rating scale (NRS) was used to evaluate pain in bone and joint disease in the past 24 h. Scores ranged from 0 to 10, with higher scores indicating more severe pain. A score of 0 represents no pain, 1–3 means mild, 4–6 moderate and 7–10 severe. This scale was validated in patients' own language and was illustrated in fitting reliability and validity.

#### 2.2.3 Hospital Anxiety and Depression Scale

The hospital anxiety and depression scale was compiled by Zigmond et al. (Civilotti et al. 2020) and has been widely used to assess anxiety and depression in Chinese patients (Ye and Xu 1993). This is made available in two subscales, 7 items of anxiety (Hospital Anxiety, HA) and 7 items of depression (Hospital Depression, HD) respectively. A 4‐point Likert scale was used to score each item ranging from 0 to 4, with a total score between 0 and 21. A score between 0 and 7 is negative, 8 and10 is mild, 11 and 14 is moderate and 15 and 21 is severe. The Cronbach's α coefficient of the Chinese version was 0.879 (Sun et al. 2017), and our result was 0.843, showing acceptable reliability and validity.

#### 2.2.4 Fatigue Scale‐14

The fatigue scale‐14 (FS‐14) was used to measure fatigue in KOA patients. The scale was compiled by Trudie Chalder and Berelowitz (Chalder et al. 1993), translated into Chinese version by Gao et al. (Gao et al. 2014). It is separated into two categories with 14 items; the first category is physical fatigue, including items 1–8, and the second category is mental fatigue, containing items 9–14 Notably, apart from the reverse score of items 10, 13 and 14, the remaining are scored with 1 point if the answer is ‘yes’ and 0 points if ‘no’. The total score is 14, with a higher score indicating more serious fatigue. The Cronbach's α in this investigation was 0.853.

#### 2.2.5 Self‐Rating Scale of Sleep

The self‐rating scale of sleep (SRSS) was compiled by Chinese scholar Li (Li 2012), and is used to estimate sleep status during the last month. It includes 10 items, each scored on a 5‐point Likert scale ranging from 0 to 5. The minimum score is 10 (basically no sleep problems) and the highest is 50 (serious sleep problems). The higher the score, the more serious the sleep problems. The Cronbach's α in this study was 0.882.

#### 2.2.6 Self‐Efficacy for Managing Chronic Disease

This scale was compiled by Lorig (Lorig et al. 2001) and sinicised by Fu et al. (Fu et al. 2003). The scale includes 6 items, dividing each item into 1 (no confidence at all) to 10 (full confidence) grades (Chalder et al. 1993). Higher scores indicate higher self‐efficacy. According to average scores, scores less than 4 indicate lower self‐efficacy, 4–7 as in the middle, and over 7 as high self‐efficacy. The reliability of the scale in this study was 0.893.

#### 2.2.7 The MOS 36‐Item Short‐Form Health Survey

We used the MOS 36‐Item Short‐Form Health Survey (SF‐36) to estimate global measures of physical, mental functioning and well‐being of KOA patients, with scores ranging from 0 to 100 (Yarlas et al. 2018). Jiang et al. translate it into Chinese version (Jiang and Li 2013). It is an eight‐subscale, 36‐item self‐reported questionnaire, containing 10 items to assess physical function, 4 items of role limitations for physical health problems, 2 items bodily pain, 5 items general health, 4 items vitality, 2 items social functioning, 3 items of role limitations due to emotional health problems and 5 items for mental health. Further, the higher the score, the better the QoL (Bunevicius 2017). The Cronbach's α in our research was 0.923.

### 2.3 Statistical Analysis

Descriptive statistics with KOA objects were calculated using frequency, percentage, mean and standard deviation (SD).

The five symptoms of pain, anxiety, depression, fatigue and sleep in KOA participants were clustered by LPA based on the Akaike information criterion (AIC), Bayesian information criterion (BIC), entropy, Lo–Mendell–Rubin (LMR) and Bootstrapped (BLRT). AIC was used to judge the quality of the model fitting by comparing the difference between the expected value and the actual value; the smaller the AIC, the better the model fit (Ling et al. 2015). BIC is similar to AIC (Xia et al. 2018). Entropy was used to evaluate the classification error rate of the model (ranging from 0 to 1); a value closer to 1 indicates accuracy, entropy < 0.6 means at least 20% of individuals had classification errors and entropy = 0.8 indicates that the classification accuracy is more than 90% (Wang and Bi 2018). If entropy is > 0.8, the model classification accuracy is better (DeMartini and Fucito 2014). BLRT and LMR were used to compare the fitting effect between the k‐th and the k‐1 category model. If *p* < 0.05, the fitting effect of the k‐th category model is better than that of the k‐1 category model (Abdi 2010). AIC, BIC, entropy, LMR and BLRT were used together to choose the final clustering, and sensitivity analyses were undertaken to investigate the robustness of the clustering.

Also, univariate analysis and chi‐square test were used to explore the differences in demographic and clinical characteristics of the LC in KOA patients, and then, only those covariates that were significantly associated with symptom group in the univariate analyses were included in the multinomial logistic regression model. The differences between LC and QOL in KOA patients were compared by one‐way ANOVA and multivariate linear regression analysis (the QOL was normality distributed and meet the assumptions of ANOVA/linear regression after checked). SPSS 25.0(IBM Corp, Armonk, NY) and Mplus 7.0 (Muthén & Muthén, USA) were used for statistical analysis. A *p*‐value of < 0.05 was considered statistically significant.

## 3 Results

### 3.1 Characteristics of Latent Classes in Patients With KOA

In our study, 242 participants finished our questionnaire, including 12 questionnaires with more than 10% missing data, and finally 230 valid questionnaires were collected. Table 1 presents the demographic and clinical characteristics of all participants.

**TABLE 1:** General data description of research objects.

Variable	Category	*n*(%) or mean ± SD
Gender	Male	52 (22.6)
	Female	178 (77.4)
Age (years)		64.54 ± 7.59
Residence	Rural	149 (64.8)
	Town	81 (35.2)
Education	Primary school and/or below	114 (49.6)
	Junior middle school	70 (30.4)
	Senior high school and/or above	46 (20.0)
Marital status	The spouse is alive	205 (89.1)
	Unmarried/widowed/divorced	25 (10.9)
Economic situation	Better	79 (34.3)
	General	60 (26.1)
	Poor	91 (39.6)
Living style	Lived with one's family	207 (90.0)
	Live alone	23 (10.0)
BMI (kg/m^2^)	Underweight (< 18.5)	1 (0.4)
	Normal (18.5 ~ 22.9)	29 (12.6)
	Overweight (23 ~ 24.9)	98 (42.6)
	Obesity (≥ 25)	102 (44.4)
Position of the knee	One	127 (55.2)
	Both	103 (44.8)
Disease course (years)	< 1	17 (7.4)
	1 ~ 5	94 (40.9)
	5 ~ 10	53 (23.0)
	> 10	66 (28.7)
Chronic complications (species)	None	82 (35.7)
	One	95 (41.3)
	≥ Two	53 (23.0)
Imaging grading	II	44 (19.1)
	III	130 (56.6)
	IV	56 (24.3)
Self‐efficacy	Low	62 (27.0)
	Middle	109 (47.3)
	High	59 (25.7)
Negative life events	Yes	69 (30.0)
	No	161 (70.0)

*Note:* BMI (kg/m2) = body mass index, Underweight (< 18.5), Normal (18.5 ~ 22.9), Overweight (23 ~ 24.9), Obesity (≥ 25); Imaging grading, 0 = Normal; I = suspected narrowing of joint space, possibly osteophyte; II = obvious osteophyte, suspected narrowing of joint space; III = moderate osteophyte, narrowing of the joint space with sclerosing changes; IV = serious osteophytes, obvious narrowing of joint space, severe sclerosis lesions and obvious sclerotic shape.

### 3.2 Classification and Description of the Best‐Fitting Latent Class

As listed in Table 2, LPA calculated pain, anxiety, depression, fatigue and sleep in KOA patients. We attempted to estimate between 1 and 5 groups, and compared the groups on the AIC, BIC, entropy, LMR and BLRT to choose the final model. First, Model 1 is fundamental. Regarding Model 2, AIC and BIC decreased sharply, entropy was 0.805, and LMR and BLRT were < 0.001. After extending the model class number from 2 to 3 in Model 3, AIC and BIC still showed a decreasing trend, entropy was 0.801 and LMR and BLRT were < 0.05. After increasing the model class number to 4 in Model 4, AIC was inadequate, BIC increased, entropy was reduced and BLRT remained < 0.001, yet LMR was > 0.05, proving that Model 4 was worse than Model 3. The class number was increased to 5 in Model 5, which altered the fitting indexes similarly to Model 4. In summary, Model 3 was the best fitting model. Meanwhile, the average attribution likelihood of the three categories was over 85%, including LC1 (95.0%), LC2 (87.9%) and LC3 (86.8%).

**TABLE 2:** PLA fitting information of latent class in KOA patients.

Model	Log‐likelihood	AIC	BIC	Entropy	LMR	BLRT	Categorical Probability
M1	−3055.02	6130.05	6164.43	—	—	—	—
M2	−2944.70	5921.40	5976.41	0.805	< 0.001	< 0.001	0.58
							0.42
M3	−2913.50	5871.00	5946.64	0.801	0.034	< 0.001	0.56
							0.24
							0.20
M4	−2899.56	5855.12	5951.39	0.738	0.426	< 0.001	0.30
							0.27
							0.21
							0.22
M5	−2886.90	5841.80	5958.70	0.774	0.354	< 0.001	0.32
							0.09
							0.21
							0.23
							0.15

Abbreviations: AIC, Akaike information criterion; BIC, Bayesian information criterion; BLRT, Bootstrapped; LMR, Lo–Mendell–Rubin.

**FIGURE 1:**
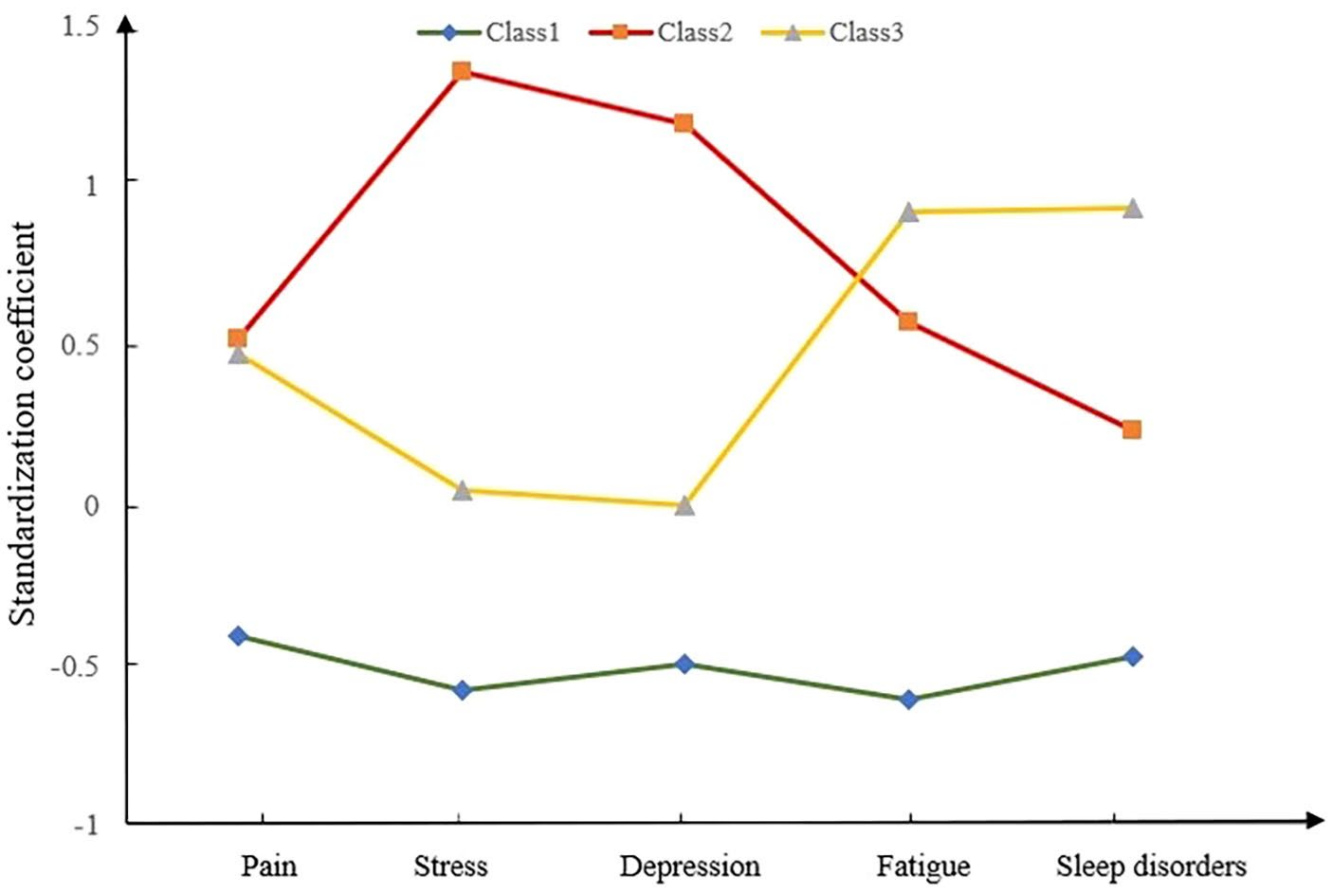
Latent class of symptom clusters in patients with KOA.

As shown in Figure 1, we identified three symptom clusters in the patients (LC1, LC2 and LC3). LC1 showed a relatively lower score than others, and it was named the ‘low symptom class’, accounting for 56% of all KOA patients. Compared with LC1, LC2 and LC3 had medium and top scores for pain, anxiety, depression, fatigue and sleep. However, LC2 was fairly higher than LC3 in anxiety, depression and psychological distress, while fatigue and sleep symptoms were rather low; thus, LC2 was considered the ‘high psychological disorder class’, including 24% of the samples. Notably, the scores of fatigue and sleep disorders in LC3 were noticeably higher than those in LC1 and LC2, and so it was considered the ‘high fatigue and sleep disorder class’, including 20% of all participants.

### 3.3 Factors Associated With Symptom Clusters

Table 3 shows the differences between demographic and clinical characteristics and the latent class of symptom clusters with KOA that were considered invalid by univariate analysis. In addition, the results revealed significant differences in age, education, economic status, chronic complications, imaging grade, self‐efficacy and experience negative life events between clusters (*p* = 0.001, 0.008, 0.007, 0.002, < 0.001, < 0.001 and 0.043 respectively). We then included these variables in the logistic model, as shown in Table 4. The age of KOA patients appeared in LC3 (OR = 1.105, 95% CI = [1.040, 1.173]), and regarding education, the junior middle school occurred in LC3 (OR = 4.133, 95% CI = [1.250, 13.664]). Moreover, in contrast to improved economic status, LC2 includes those who are average or poor (OR = 3.292, 95% CI = [1.106, 9.801]). Regarding having two or more chronic complications, C1 may include none (OR = 0.713, 95% CI = [0.238, 2.136]). Furthermore, the higher knee imaging grade was found in LC2 and LC3 (OR = 0.249, 95% CI = [0.067, 0.092]; OR = 0.379, 95% CI = [0.145, 0.991] respectively). With no adverse life events during the past year for reference, this probably emerged in LC2 (OR = 2.517, 95% CI = [1.103, 5.743]). Additionally, lower self‐efficacy was in LC2 and LC3 (OR = 60.281, 95% CI = [11.753, 309.182]; OR = 4.786, 95% CI = [1.523, 15.043] respectively).

**TABLE 3:** Characteristics of three latent classes in patients with KOA (univariate analysis).

	LC1	LC2	LC3	*F/χ* ^2^	*P*	*P*_(LC1 vs. LC2)_	*P*_(LC1 vs. LC3)_	*P*_(LC2 vs. LC3)_
Variables	Mean ± SD	Mean ± SD	Mean ± SD					
Age (years)	63.63 ± 8.15	63.50 ± 6.94	68.30 ± 5.37	7.496	0.001	0.911	< 0.001	0.001
	*n* (%)	*n* (%)	*n* (%)					
Education				13.783	0.008	0.107	0.008	0.128
Primary school and/or below	62 (48.4)	33 (58.9)	19 (41.3)					
Junior middle school	32 (25.0)	16 (28.6)	22 (47.8)					
Senior high school and/or above	34 (26.6)	7 (12.5)	5 (10.9)					
Economic situation				14.063	0.007	0.001	0.716	0.001
Better	54 (42.2)	8 (14.3)	17 (37.0)					
General	29 (22.7)	18 (32.1)	13 (28.2)					
Poor	45 (35.1)	30 (53.6)	16 (34.8)					
Chronic complications (species)				16.830	0.002	0.098	0.001	0.119
None	58 (45.3)	16 (28.6)	8 (17.4)					
One	47 (36.7)	28 (50.0)	20 (43.5)					
≥ Two	23 (18.0)	12 (21.4)	18 (39.1)					
Imaging grading				20.562	< 0.001	< 0.001	0.028	0.158
II	27 (21.1)	6 (10.7)	11 (23.9)					
III	83 (64.8)	26 (46.4)	21 (45.7)					
IV	18 (14.1)	24 (42.9)	14 (30.4)					
Self‐efficacy				54.094	< 0.001	< 0.001	0.001	0.002
Low	13 (10.2)	33 (58.9)	16 (34.8)					
Middle	70 (54.7)	21 (37.5)	18 (39.1)					
High	45 (35.1)	2 (3.6)	12 (26.1)					
Negative life events				6.295	0.043	0.085	0.021	0.563
Yes	30 (23.4)	20 (35.7)	19 (41.3)					
No	98 (76.6)	36 (64.3)	27 (58.7)					

Abbreviations: C, Control group; LC1, Low symptom class; LC2, High psychological disorder class; LC3, High fatigue with sleep disorder class.

**TABLE 4:** Characteristics of three latent classes in patients with KOA (multinomial logistic regression).

Variables	LC1 vs. LC2^a^				LC1 vs. LC3^a^				LC2 vs. LC3^b^			
	*B*	*p*	OR	95% CI	*B*	*P*	OR	95% CI	*B*	*p*	OR	95% CI
Intercept	−3.814	0.075	—	—	−8.010	< 0.001	—	—	4.196	0.103	—	—
Age (years)	0.004	0.899	1.004	[0.950,1.060]	0.099	0.001	1.105	[1.040,1.173]	−0.096	0.005	0.909	[0.850,0.971]
Education												
Senior high school and/or above	C				C				C			
Junior middle school	0.432	0.490	1.540	[0.452,5.249]	1.419	0.020	4.133	[1.250,13.664]	−0.987	0.194	0.373	[0.084,1.654]
Primary school and/or below	0.145	0.811	1.157	[0.352,3.805]	0.622	0.322	1.863	[0.543,6.391]	−0.447	0.533	0.621	[0.139,2.776]
Economic situation												
Better	C				C				C			
General	1.191	0.035	3.415	[1.088,10.714]	0.563	0.300	1.756	[0.605,5.096]	0.655	0.311	1.944	[0.537,7.044]
Poor	1.228	0.032	3.292	[1.106,9.801]	0.267	0.604	1.307	[0.475,3.593]	0.924	0.141	2.520	[0.736,8.624]
Chronic complications (species)												
None	−0.338	0.546	0.713	[0.238,2.136]	−1.584	0.004	0.205	[0.070,0.603]	1.246	0.047	3.476	[1.108,11.870]
One	0.646	0.237	1.909	[0.654,5.571]	−0.720	0.154	0.487	[0.181,1.309]	1.366	0.017	3.921	[1.279,12.023]
≥ Two	C				C				C			
Imaging grading												
II	−1.391	0.038	0.249	[0.067,0.927]	−0.329	0.573	0.720	[0.229,2.263]	−1.062	0.127	0.346	[0.088,1.354]
III	−1.265	0.007	0.282	[0.113,0.706]	−0.969	0.048	0.379	[0.145,0.991]	−0.296	0.557	0.744	[0.277,1.998]
IV	C				C				C			
Negative life events												
Yes	C				C				C			
No	0.320	0.455	1.377	[0.595,3.186]	0.923	0.028	2.517	[1.103,5.743]	−0.603	0.196	0.547	[0.219,1.366]
Self‐efficacy												
Low	C				C				C			
Middle	2.050	0.010	7.771	[1.643,36.765]	0.268	0.585	1.308	[0.499,3.427]	1.782	0.039	5.964	[1.099,32.141]

Abbreviations: C, control group; LC1, low symptom class; LC2, high psychological disorder class; LC3, high fatigue with sleep disorder class.

^a^ indicates that LC1 is used as a reference in logistic regression.

^b^ indicates that LC3 is used as a reference in logistic regression.

To summarise, being younger, having obtained a senior high school education and above, improved economic conditions, lack of chronic complications, lower knee imaging grade, no experience of negative life events during the past year, and higher self‐efficacy in KOA were assigned to LC1. Additionally, LC2 included those who are younger, have a poor economic status, complicated with chronic diseases, with higher knee imaging grade, experienced negative life events during the past year and had lower self‐efficacy. Noticeably, those who were older, less educated, complicated with chronic diseases, had higher knee joint imaging grade, experienced negative life events during the past year and had lower self‐efficacy were found in LC3.

### 3.4 Relationship Between Latent Class of Symptom Clusters and QoL

Table 5 reveals the results of multiple linear analyses. Statistical analysis revealed that the scores of LC2 and LC3 (*B* = 20.722, 95% CI = [3.670, 37.774]; *B* = −10.906, 95% CI = [−17.609, −4.203] respectively) were lower than those of LC1. In addition, a statistical difference was found between LC2 and LC3 (Model 1, Table 5), indicating that it explained 25.30% of the variation in QoL among KOA patients. After recalculating the demographic and clinical variables (Model 2, Table 5), the scores of QoL in LC2 and LC3 were still lower than LC1 (*B* = 11.049, 95% CI = [2.621, 19.477]; *B* = −7.622, 95% CI = [−11.777,‐3.467] respectively). No significant difference was noted between LC2 and LC3 (*B* = 3.786, 95% CI = [2.022, 5.550]). In conclusion, three LCs explained 39.2% of the variation in QoL among KOA patients.

**TABLE 5:** The relationship between three latent classes and QoL (multiple linear analysis).

Variables	Model 1			Model 2		
	*B*	*t*	*p*	*B*	*t*	*p*
LC1 vs. LC2^a^	−20.722	−8.721	< 0.001	−11.409	−4.353	< 0.001
LC1 vs. LC3^a^	−10.906	−4.278	< 0.001	−7.622	−2.916	0.004
LC2 vs. LC3^b^	9.816	3.326	0.001	3.786	1.296	0.197
Adjusted *R* ^2^	25.30%			39.20%		

*Note:* The superscript of a indicates that LC1 is used as a reference in Logistic regression; b represents that LC3 is used as a reference in Logistic regression. Model 1 = no control variables; Model 2 = controls variables such as age, sex, education, economic status, course of a disease, chronic complications, knee joint imaging grading, negative life events and self‐efficacy.

Abbreviations: C, Control group; LC1, Low symptom class; LC2, High psychological disorder class; LC3, High fatigue with sleep disorder class.

## 4 Discussion

In the present study, we identified three LCs based on pain, anxiety, depression, fatigue and sleep disorders using the LPA among patients with KOA. The three LCs were labelled as follows: LC1, ‘low symptom class’; LC2, ‘high psychological disorder class’; and LC3, ‘high fatigue and sleep disorder class’, which accounted for 56%, 24% and 20% of all participants respectively. However, compared with Jenkins et al., several symptom cluster differences existed, and this previous study produced two groups based on cluster analysis of 75 participants with KOA, called the ‘low pain and high depression’ and ‘symptom balance’ groups (Jenkins and McCoy 2015). The differences could be attributable to several occurrences. First, anxiety and sleep indicators were included in our study. Second, there may be differences in cultural regions, population quality and medical care, which would account for various feelings of symptoms. Third, the sample size of Jenkins et al. was limited, suggesting a lack of appropriate representation. Fourth, in contrast to classification methods, Jenkins applied cluster analysis, while the LPA employed in this research selected different statistical analysis methods and, thus, the symptom cluster results would be altered (Shi and Li 2018). Also, the results of cluster analysis are subjective, while the LPA will associate the symptoms with the object, and the grouping result is more worthwhile to explore a symptom cluster grouping (Ji et al. 2017).

According to our results, older patients with KOA are likely to be categorised in the latent class of ‘high fatigue and sleep disorder class’. Previous study have shown that aging, the capacity of organ function, self‐care and loss of social adaptability exacerbate fatigue symptoms (Park et al. 2019). In addition, the pineal gland weakly secretes melatonin, and this produces sleep problems, such as limiting sleep time, unstable sleep and early awakening. Convincingly, many researchers have confirmed the common influence and causal relationship between fatigue and sleep (Fertelli and Tuncay 2019). Further, individuals with KOA who have also obtained a higher education have an increased likelihood to gain awareness of health care through media networks, books and other communication channels; thus, this lessens the confusing symptoms caused by the disease (Wu et al. 2016). Therefore, the more educated an older adult is, the more likely they are to be classified in the ‘low symptom class’. Regarding economic conditions, most studies have proven that mental illness varies depending on the country, region and individual (Jenkins et al. 2008; Mackenbach et al. 2008). Moreover, the sample that meets the criteria of relative poverty has a 1.5–2.0 times higher risk of anxiety, depression and commonalities, as suggested by the World Organisation for Economic Cooperation and Development. This may explain that KOA patients with poor financial status may be classified in the ‘psychological disorder group’.

Concerning the clinical characteristics of the KOA participants in the current study, those with fewer chronic complications might fit into the ‘low symptom class’. In addition, Liu's study demonstrated that compared with middle‐aged people who did not experience chronic diseases, their occupation directly increased the risk of experiencing anxiety and depression symptoms (Liu et al. 2010). Further, Several studies showed that the amount of chronic diseases and pain can indirectly affect depression through the individual's view or outlook of their own health and sleep duration of the path analysis model (Ho et al. 2020; Zhang et al. 2018). Resulting from chronic complications due to hypertension, diabetes and coronary heart disease in our study, the use of antihypertensive diuretics was common, which leads to developing nocturnal urination, and this in turn affects sleep rhythm. In addition, antihypertensive diuretics could produce side effects such as dizziness, palpitation, fatigue and so on, which further impact sleep (Liu et al. 2017). Moreover, imaging grading becomes a vital index for the diagnosis of KOA, which controls the diagnosis decision to a certain extent. According to Wang's results (Wang et al. 2015), the imaging grade of the patellofemoral joint was positively correlated with pain, and this could directly affect one's psychological experience and sleep, resulting in anxiety, depression and sleep disorders (Wang et al. 2015). Thus, a higher imaging grade indicates more concerns about knee joint stiffness, deformation, daily disorder and ease of tiredness. Therefore, a higher imaging grade was found in the ‘high psychological disorder class’ and ‘high fatigue and sleep disorder class’.

Self‐efficacy and the occurrence of negative life events were individual characteristics considered in this study, which improves the capacity to associate with latent classes of symptom clusters among KOA cases. To our knowledge, self‐efficacy symbolises an individual who believes in their ability to achieve specific goals (Lev 1997). Current research showed that high self‐efficacy is fundamental to heighten the capacity of self‐management, enrich behaviour and preserve health (Haas 2000). Also, self‐efficacy has a regulatory role in one's ability to overcome physical symptoms, as shown in a longitudinal study with KOA patients (Sperber et al. 2014). Therefore, higher self‐efficacy would be found in the ‘low symptom class’. Conversely, experiencing a negative life might heighten an individual's psychological stress and shorten their satisfying life (Marum et al. 2014). Further, Zhang found that compared with healthy older adults, those with chronic diseases tended to adopt pessimistic outlooks when encountering negative events, which created depressive symptoms (Zhang et al. 2017). Thus, a person with such conditions would presumably be included in the ‘psychological disorder group’.

QoL is comprehensive since it includes the physical, psychological and social roles of individuals (Sun et al. 2015). Distinctively, our findings showed that the QoL of KOA patients in LC2 and LC3 was inferior to that of patients in LC1. As is well known, pain is listed as the fifth vital sign. A patient repeatedly experiencing a sustained disease course, recurrent chronic pain and serious sleep trouble will weaken in their physical resistance and self‐care ability. Furthermore, these factors were negatively correlated with QoL (Pope 2020; Srivastava et al. 2017). Consequently, sleep disorders caused by pain will contribute to anxiety and depression, thus exacerbating sleep disorders. However, unnatural sleep will account for fatigue, creating a vicious cycle of multiple symptoms that affect the social ability and QoL in KOA patients (Matcham et al. 2014; Wang et al. 2019). After the inclusion of control variables, there was no statistically significant difference in QoL between the LC2 and LC3. This suggests that when groups have multiple symptoms, the difference in QoL cannot be explained by the symptom group itself, and the newly added control variables are the main source of variation.

### 4.1 Limitations

While these discoveries are noteworthy, this study had several limitations to take into consideration. The impact of the cross‐sectional study design remains to be determined, such as the inability to detect and associate with the longitudinal changes of symptoms among KOA patients. Another cohort study is warranted in the future to better understand these findings. Further, most subjects were older adults, and the data were mainly collected by subjective self‐administered questionnaires, which may be biased to a certain extent. Therefore, objective signals (such as the blood routine examination) to measure pain, anxiety, depression, fatigue and sleep disorders should be adopted in future studies. Moreover, our participants were primarily recruited in outpatient clinics and wards in two tertiary hospitals by convenient sampling, and the representation of the population may not be appropriate compared with random sampling. Additionally, most participants selected had diseases in the active stage, which might account for a higher frequency of symptoms. Therefore, a large, multicentre study with random sampling is needed to further explore the latent class of cluster symptoms in patients with KOA over time.

## 5 Conclusions

The study identified LCs according to pain, anxiety, depression, fatigue and sleep disorder symptoms among patients with KOA, providing a foundation for further determining whether the clusters vary over time or along disease and treatment trajectory and what their possible synergistic effects are on KOA and associated QoL (Wang and Bi 2018). Symptom cluster identification can serve as the basis for tailored interventions for KOA to improve QoL and reduce healthcare utilisation and associated costs.

## Author Contributions

**Guang‐Zhao Li**, **Rong‐Jian Ji** and **Cui Ping Xu** contributed equally to the article in conceptualising, methodology and writing the original manuscript and are co‐first authors; **Li‐Juan Yang:** data curation and formal analysis; **Paulo Moreira:** formal analysis, visualisation, review and editing.

## Ethics Statement

This study has been approved by the Ethics Committee of Shandong Provincial Hospital Affiliated to Shandong First Medical University (SWYX: 2019‐189).

## Consent

The authors have nothing to report.

## Conflicts of Interest

The authors declare no conflicts of interest.

## Data Availability

The data that support the findings of this study are available from the corresponding author upon reasonable request.
